# Restricted Kinematically Aligned Total Knee Arthroplasty Following Failed Oxford Unicompartmental Knee Arthroplasty

**DOI:** 10.7759/cureus.45104

**Published:** 2023-09-12

**Authors:** Takuma Hayashi, Takafumi Hiranaka, Takaaki Fujishiro, Koji Okamoto, Motoki Koide

**Affiliations:** 1 Orthopaedic Surgery and Joint Surgery Centre, Takatsuki General Hospital, Takatsuki, JPN

**Keywords:** kinematically aligned total knee arthroplasty, technique, revision arthroplasty, failed unicompartmental knee arthroplasty, knee

## Abstract

In this report, we describe how to revise a failed Oxford unicompartmental knee arthroplasty to kinematically aligned total knee arthroplasty (TKA). Its benefits are the maintenance of the native joint line along with the avoidance of supplemental parts, such as metal augments and stems. This can be applied to patients whose medial tibial cortex is well preserved. The distal cutting plane and rotation alignment are decided before the removal of the femoral component. The tibial cutting plane is up to 12 mm below the lateral joint surface and the varus is up to 5° below the extramedullary rod. Eventually, the native joint line and alignment along with the soft tissue envelope can be well maintained, similar to the restricted kinematically aligned TKA.

## Introduction

Revision total knee arthroplasty (TKA) such as a fracture after failed unicompartmental knee arthroplasty (UKA) is a challenging procedure [1,2]. Mechanical alignment TKA (MA-TKA) has been used with augments, wedges, and stems to restore bone loss and stabilize the component for better clinical results and implant survivorship [3]. However, ligament balancing may be difficult due to uneven bone loss and imbalanced flexion and extension gaps. Moreover, the use of supplemental parts to compensate for the bone loss and facilitate component stability results in prolonged operation time, filled bone gaps, and more complicated procedures.

Kinematically aligned TKA (KA-TKA) is a reasonable alteration for revision TKA after failed Oxford UKA (OUKA) [4]. Most surrounding soft tissues can be preserved and are well balanced, and because the Oxford UKA is a resurfacing surgery, the native joint surface can be preserved. Femoral bone cutting can be, therefore, made with reference to the component surface. A further benefit is that the tibia is cut in varus which can compensate for the bone loss of the medial tibial plateau, making supplemental parts unnecessary. Despite the potential efficacy of the KA-TKA after failed UKA, the details have not been clearly described [5,6]. Moreover, revision KA-TKA after Oxford mobile-bearing UKA has not been reported. We present the details of how we perform the procedure in this technical report.

## Technical report

Patient selection

**Figure 1:**
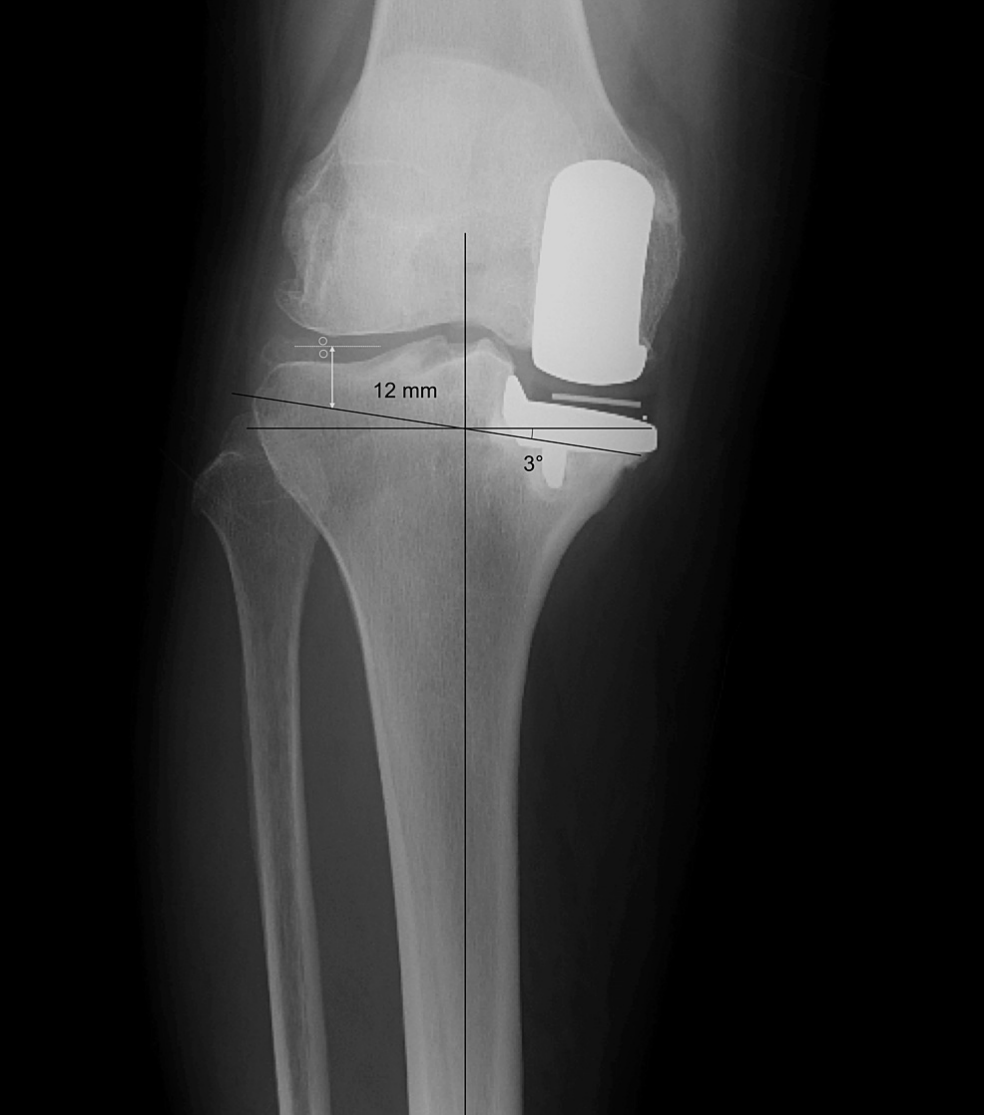
Preoperative radiographic evaluation. The estimated cutting line is drawn from the highest point of the intact medial tibial cortex with 3° varus forming a perpendicular line of the tibial axis. This line should be <12 mm from the lateral joint line.

Patient selection is based on medial cortex continuity and retention of the medial collateral ligament. The proximal end of the intact medial cortex would be the medial end of the cutting surface. If pre-UKA radiography is available, the medial proximal tibial angle is measured and replicated on the radiograph before re-revision. Similar to restricted KA-TKA, we limit varus inclination up to 5° [6]. The lateral cutting thickness is also measured (Figure 1); if it is >12 mm, we recommend avoiding KA-TKA. If the tibial bone loss is especially great, such as by tibial fracture, we recommend MA-TKA with stems and augments.

Procedure

**Figure 2:**
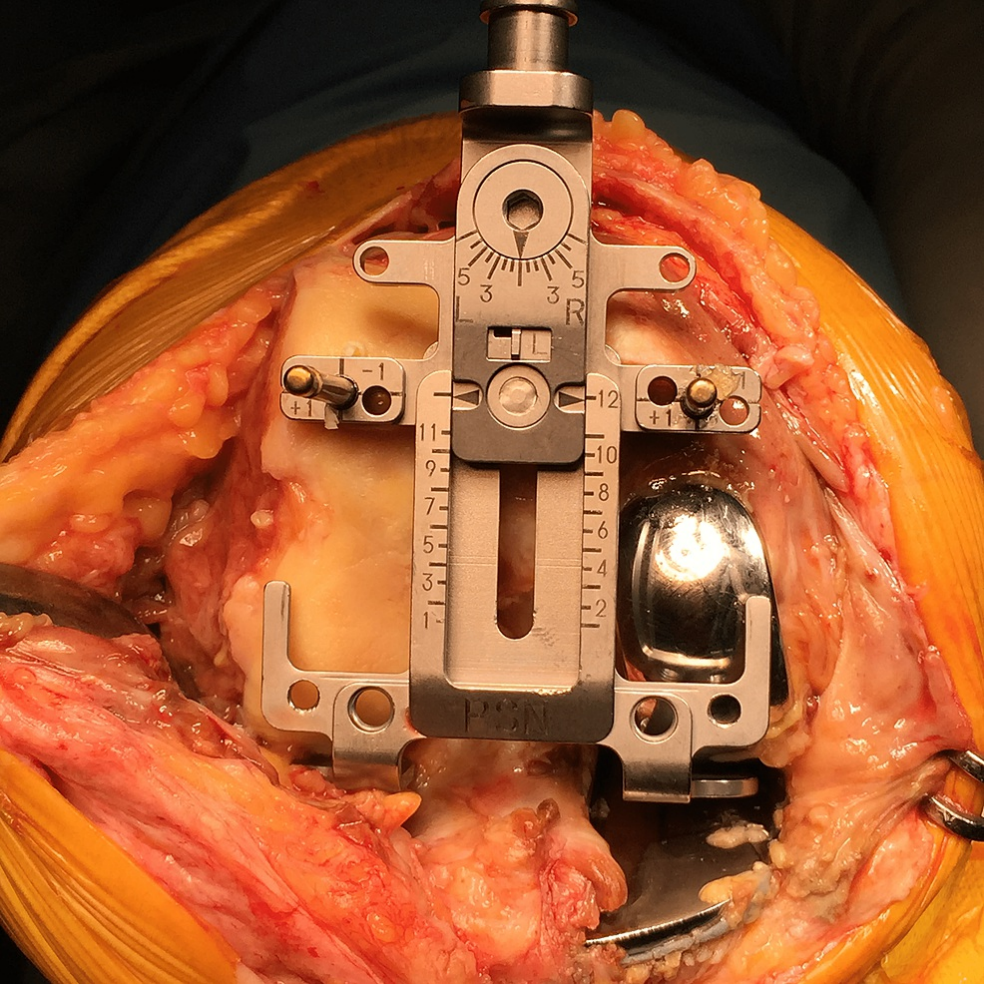
The rotation alignment of the femur is decided to place the sites of the sizer and pin insertion.

**Figure 3:**
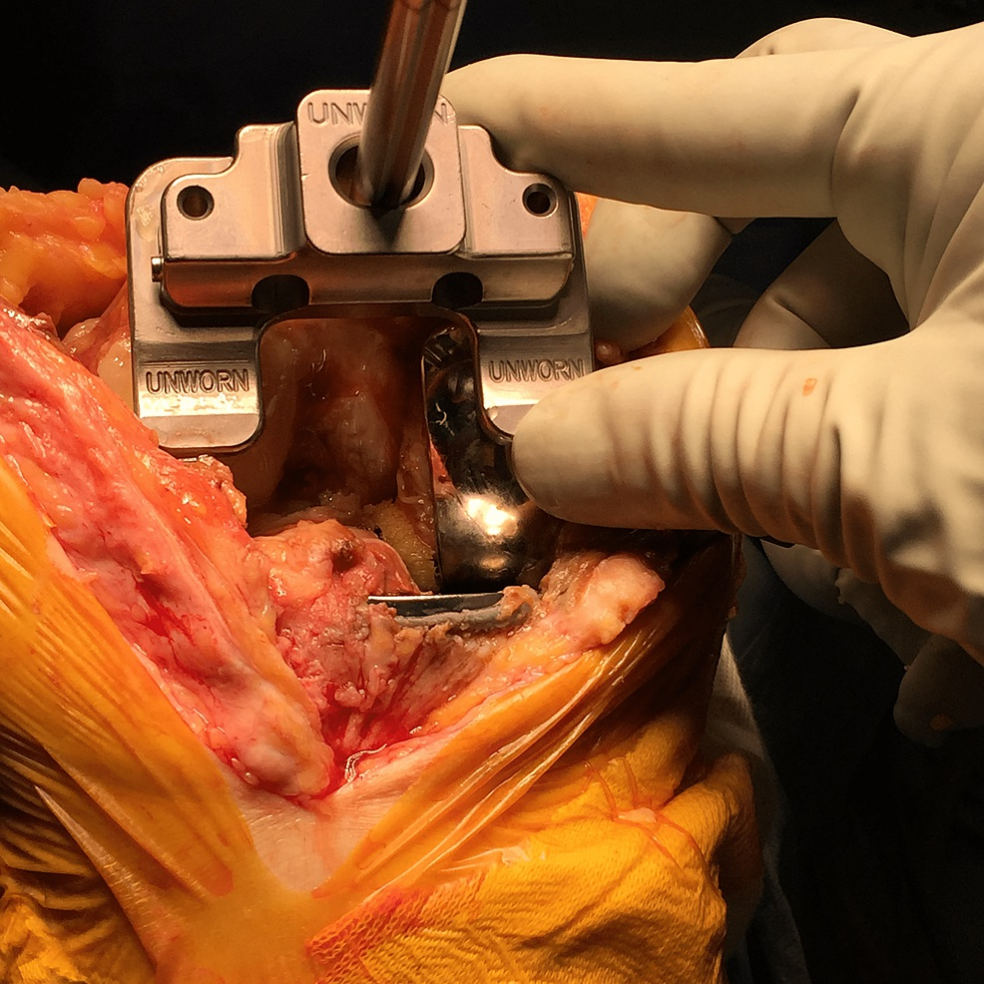
The distal femoral cutting plane is decided by placing the “unworn-unworn” paddle on the distal surface.

After the arthrotomy, the femoral sizer is set on the posterior condyles before the removal of the femoral component. Pinholes are then inserted through the sizer with the rotation set at 0° respecting the posterior condylar axis (Figure 2). An entry made for the intramedullary (IM) rod is then made at the bottom of the distal femoral notch. The IM rod is inserted and the paddle is placed (unworn-unworn one) onto both the lateral femoral cartilage and the medial component surface (Figure 3). The cutting level is set at 9 mm above the joint line, the thickness of the distal femoral component.

**Figure 4:**
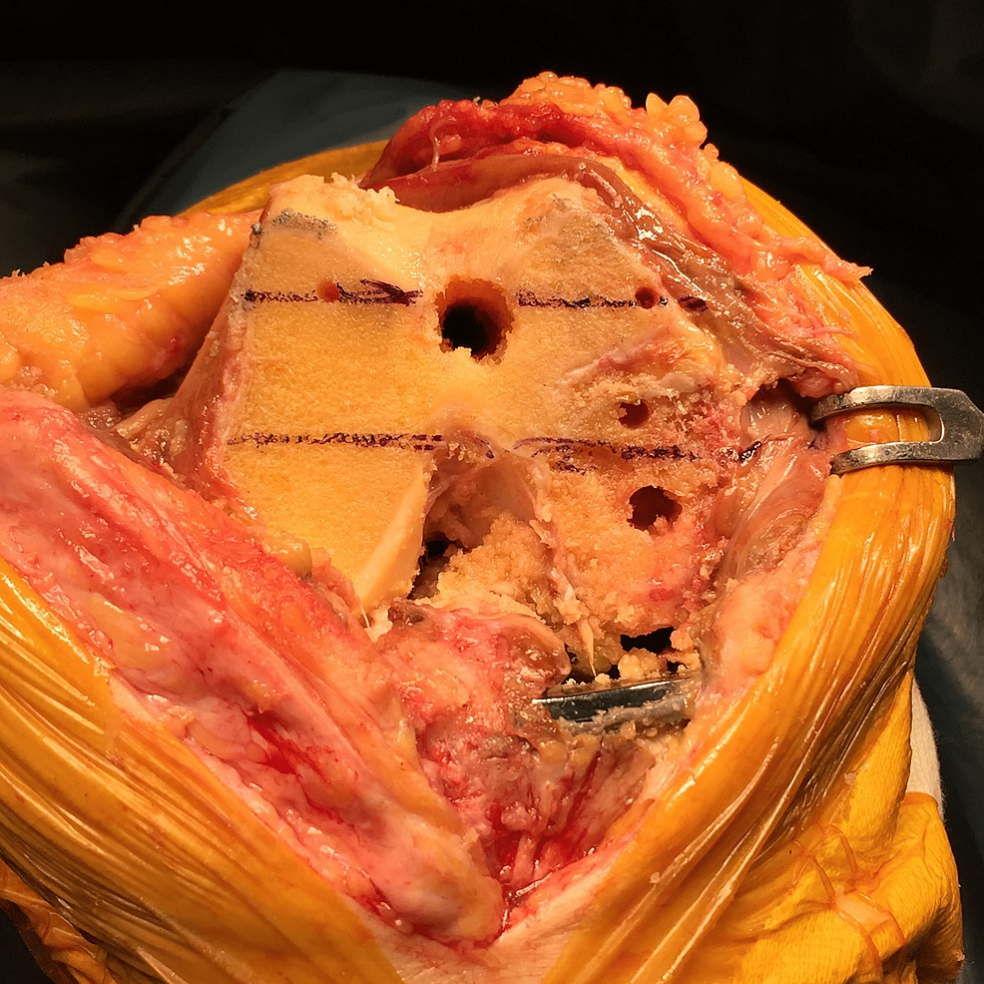
The four-in-one cutting block can be inserted into the holes, which are obvious, even after the distal cut.

After the distal femoral cutting block is set, the IM rod is removed and the femoral component is retrieved. The connection between the bone and the component is detached using a thin blade chisel around the pegs, ensuring not to damage the bone. The component surface is then hit with a hammer for loosening. The component can then be retrieved easily using the Oxford slap hammer (Zimmer Biomet, Warsaw, IN, USA) with minimum bone loss. The distal end of the femur is then cut, and the sizer is set so that the rotation is parallel to the line that connects the previously made pinholes (Figure 4).

If the tibial component is not loose, the anteromedial aspect of the tibial component is detached using a chisel. The reciprocating saw is inserted just under the tibial component and the bonding of cement and tibial component are cut lateral to the keel. The detachment medial to the keel is made by a thin chisel.

**Figure 5:**
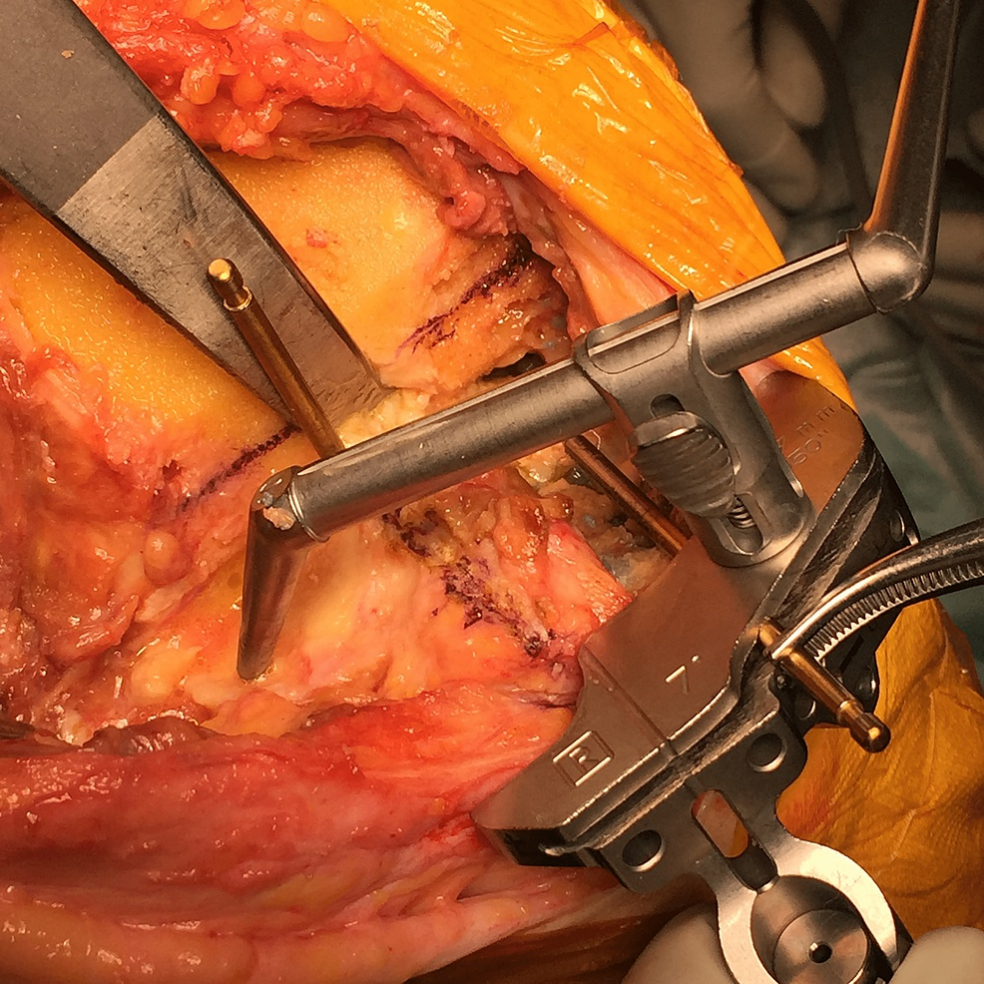
The posterior slope and cutting plane of the tibia are decided using a pin or an angel wing inserted through the cutting slot. The cutting plane should be up to 12 mm below the lateral joint line.

**Figure 6:**
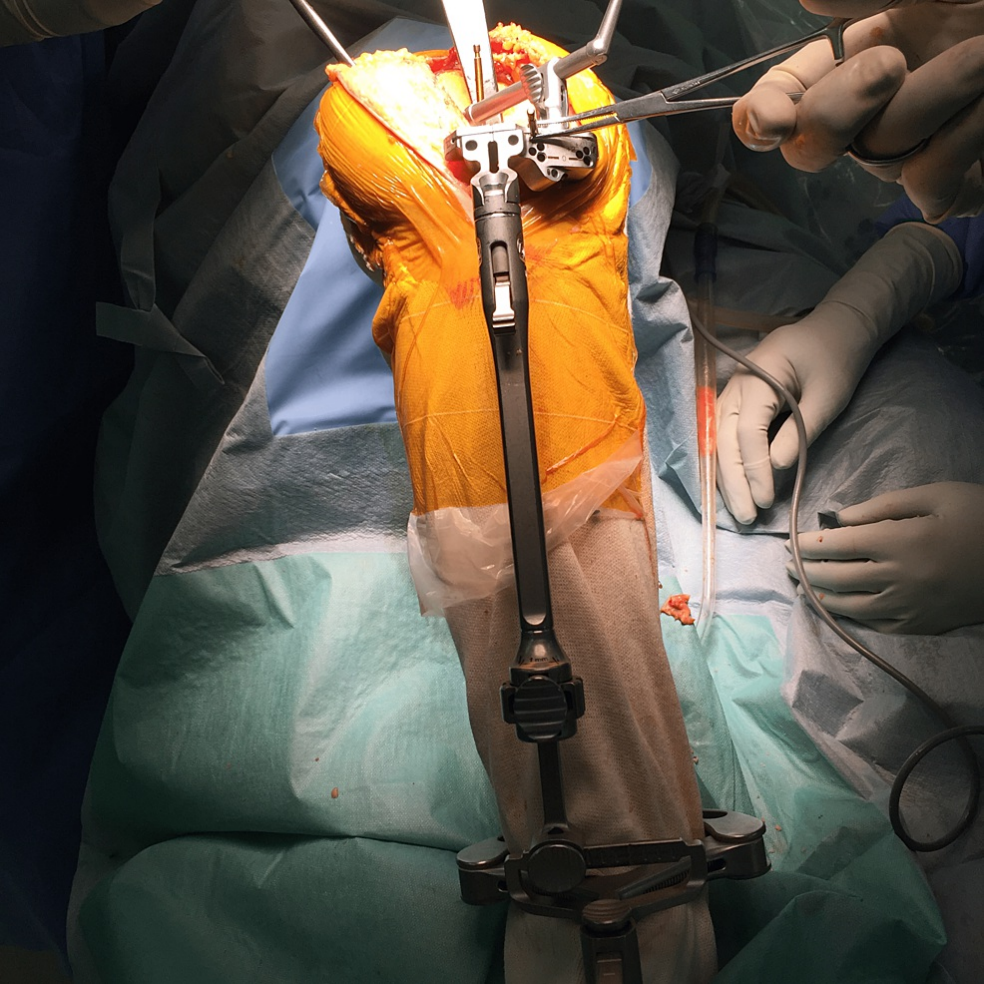
The total varus inclination can be calculated using the total length of the rod. The sliding length of the distal leg fixator should be <5°.

The posterior cruciate ligament (PCL) should be confirmed to be well preserved, and a bony island is then made to protect the insertion of the PCL on the tibia. To prevent damage to the PCL, we recommend cutting the bone using a reciprocating saw and inserting a pin into the gutter. The extramedullary tibial rod is set and an angel wing or a pin is inserted through the slot of the cutting block. The posterior slope and cutting surface of the medial tibial surface are then checked. Ideally, the cutting slot is set at just below the highest point of the medial tibial cortex and 10-12 mm below the lateral tibial articular surface (Figure 5). We recommend a maximum varus inclination of the cutting surface of 5°. In case of some extreme anatomies, such as the sliding length of the distal leg fixator of more than 5° due to fracture or necrosis, kinematic alignment should be abandoned and changed to the MA-TKA, although this should be avoided if possible (Figure 6) [7]. The tibia is cut along the cutting block slot and the flexion and extension balance are checked. If there is an imbalance, it is possible to perform a tibial recut and/or soft tissue balancing. The movement and stability of the trial components should be checked, followed by the final implantation.

Case presentation

**Figure 7:**
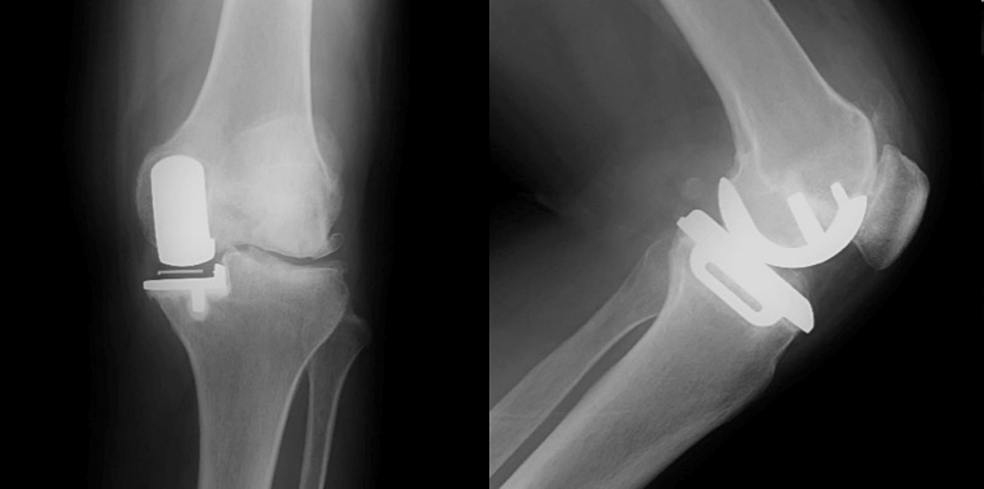
A case of a 61-year-old female with the progression of lateral osteoarthritis. Before surgery.

**Figure 8:**
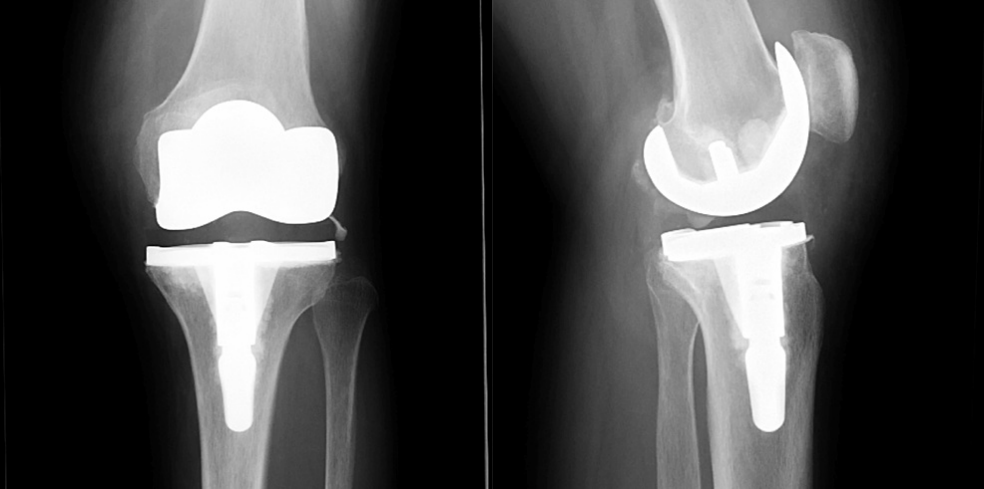
A case of a 61-year-old female with the progression of lateral osteoarthritis. After surgery.

A 61-year-old female underwent bilateral UKA with hybrid Oxford UKA (cemented tibia and uncemented femur). She had continuous pain in her left knee with motion. The progression of lateral osteoarthritis was found around the tibial component. Two years postoperatively, the KA-TKA procedure was used in revision surgery. The soft tissues, including the PCL, were well preserved and the gap balancing was stable. Radiographs taken soon after the revision showed excellent alignment (Figures 7, 8).

## Discussion

The most important benefit of the KA-TKA, even after a failed OUKA, is the maximally maintained native soft tissue balancing [8]. Unlike MA-TKA, KA-TKA can retain the native joint line with potentially minimized alteration of kinematics and ligament balance along with minimized bone cut volume [5]. The OUKA is a resurfacing surgery and the soft tissue envelope can be retained, so it is reasonable to decide the cutting plane using calipers [9]. A further benefit of this technique is that bulky supplemental parts are unnecessary.

Evaluation of the retained medial tibia cortex and the quality of the bone cutting surface is particularly important. The tibial cutting plane can be varus in KA-TKA, making the medial bone cutting level lower than MA-TKA so that it can compensate for the loss of the tibial bone [10]. Ideally, the tibial cutting line is set between 0.5 mm and 1.0 mm below the highest point of the intact medial cortex, so more than half of the length of the intact medial cortical rim along the good cutting plane is maintained. The cement-on-cement technique is beneficial in preserving bone volume as long as the cement is well-fixed to the underlying cancellous bone with sufficient cement penetration [11]. The cement around the keel slot should be removed; however, it should be filled with autologous cancellous bone chips from the resected bone fragments.

We restricted the varus angle to 5°, following the literature [12]. Cutting the tibia parallel to the femoral joint line or mimicking the original articular surface is also likely possible [13,14]. Revision surgery is a salvage surgery; however, an extreme alignment outlier should be avoided [15]. We suggest restriction is the safest option until greater varus alignment is proven to be safe. Similarly, we restricted the cutting level up to 12 mm from the lateral joint surface, assuming a 12 mm bearing. A lower cut would be preferable, but it requires a thicker bearing. Use of bearings >14 mm can reportedly increase the incidence of fracture [16]. Targeting the 12 mm bearing thickness should, therefore, be safe, considering the cutting variation.

This technique has some limitations. First, the successful revision of KA-TKA depends on the quality of the medial tibial cortex. If it has been devastatingly damaged, it cannot be compensated for, even by a tibial cut with maximal medial incline. Conversion is necessary in cases where tibial bone loss is unexpectedly larger than in the preoperative radiographic evaluation. Femoral component rotation should be changed to convert from KA-TKA (parallel to the posterior condylar axis) to MA-TKA (parallel to the transepicondylar axis). It is virtually impossible, however, because the posterior condyles are already cut and femoral component rotation has already been decided. Preparing the tibia before the femoral posterior cut would be helpful if the condition of the medial cortex is unclear. A second limitation is that it is necessary to have a preserved and well-functioning PCL owing to the use of cruciate-retaining TKA. Third, although there are some similar reports, the number of patients is small and the follow-up periods have been short [5,6,17]. A larger longer follow-up study with a greater number of patients is needed to clarify the benefits of the procedure. However, we believe this procedure is equivalent to primary KA-TKA as long as the quality of the tibial cutting surface is good and the soft tissue envelope is well preserved.

## Conclusions

Among others, KA-TKA is a good option for revision surgery after a failed UKA. The procedure can retain bone volume and soft tissue balance, avoiding bulky supplemental parts.

## References

[1] Craik JD, El Shafie SA, Singh VK, Twyman RS (2015). Revision of unicompartmental knee arthroplasty versus primary total knee arthroplasty. J Arthroplasty.

[2] El-Galaly A, Kappel A, Nielsen PT, Jensen SL (2019). Revision risk for total knee arthroplasty converted from medial unicompartmental knee arthroplasty: comparison with primary and revision arthroplasties, based on mid-term results from the Danish Knee Arthroplasty Registry. J Bone Joint Surg Am.

[3] Lewis PL, Davidson DC, Graves SE, de Steiger RN, Donnelly W, Cuthbert A (2018). Unicompartmental knee arthroplasty revision to TKA: are tibial stems and augments associated with improved survivorship?. Clin Orthop Relat Res.

[4] Lee YS, Howell SM, Won YY, Lee OS, Lee SH, Vahedi H, Teo SH (2017). Kinematic alignment is a possible alternative to mechanical alignment in total knee arthroplasty. Knee Surg Sports Traumatol Arthrosc.

[5] Shelton TJ, Gill M, Athwal G, Howell SM, Hull ML (2021). Revision of a medial UKA to a kinematic aligned TKA: comparison of operative complexity, postoperative alignment, and outcome scores to a primary TKA. J Knee Surg.

[6] Toliopoulos P, LeBlanc MA, Hutt J, Lavigne M, Desmeules F, Vendittoli PA (2016). Anatomic versus mechanically aligned total knee arthroplasty for unicompartmental knee arthroplasty revision. Open Orthop J.

[7] Hiranaka T, Tanaka T, Fujishiro T (2020). A novel technique for varus tibial cutting for Oxford unicompartmental knee arthroplasty. Clin Orthop Surg.

[8] Lustig S, Sappey-Marinier E, Fary C, Servien E, Parratte S, Batailler C (2021). Personalized alignment in total knee arthroplasty: current concepts. SICOT J.

[9] Hiranaka T, Fujishiro T, Koide M, Okamoto K (2022). Kinematic alignment bi-unicompartmental knee arthroplasty with Oxford partial knees: a technical note. Cureus.

[10] Rivière C, Iranpour F, Auvinet E, Howell S, Vendittoli PA, Cobb J, Parratte S (2017). Alignment options for total knee arthroplasty: a systematic review. Orthop Traumatol Surg Res.

[11] Tovar-Bazaga M, Sáez-Martínez D, Auñón Á, López-Oliva F, Pardos-Mayo B, Calvo E (2021). Surgical technique of a cement-on-cement removal system for hip and knee arthroplasty revision surgery. Arthroplast Today.

[12] Almaawi AM, Hutt JR, Masse V, Lavigne M, Vendittoli PA (2017). The impact of mechanical and restricted kinematic alignment on knee anatomy in total knee arthroplasty. J Arthroplasty.

[13] Hsu CE, Huang JT, Tong KM, Huang KC (2020). Total knee arthroplasty according to the original knee phenotypes with kinematic alignment surgical technique-early clinical and functional outcomes. BMC Musculoskelet Disord.

[14] Okazaki K (2022). Adopting the joint line theory for bone resection in cruciate-retaining total knee arthroplasty to prevent flexion gap tightness. Orthop Surg.

[15] Schelker BL, Nowakowski AM, Hirschmann MT (2022). What is the "safe zone" for transition of coronal alignment from systematic to a more personalised one in total knee arthroplasty? A systematic review. Knee Surg Sports Traumatol Arthrosc.

[16] Berend ME, Davis PJ, Ritter MA, Keating EM, Faris PM, Meding JB, Malinzak RA (2010). "Thicker" polyethylene bearings are associated with higher failure rates in primary total knee arthroplasty. J Arthroplasty.

[17] Asghar A, Naaz S, Narayan RK, Kumar A (2021). Does the prevalence of ossified fabella vary in knee osteoarthritis and age-related degeneration? A meta-analysis of about 11,000 knees. Cureus.

